# Research on the Linkage Mechanism between Migrant Workers Returning Home to Start Businesses and Rural Industry Revitalization Based on the Combination Prediction and Dynamic Simulation Model

**DOI:** 10.1155/2022/1848822

**Published:** 2022-09-26

**Authors:** Xiaogang Wang

**Affiliations:** College of Management, Henan University of Technology, Zhengzhou 450052, China

## Abstract

Township and rural migrant workers are returning to the business personnel's main force. Party 18 proposed a rural revitalization strategy, and the central committee of the State Council issued a series of encouraging policy measures to bring them back. The study found that migrant workers' return-home entrepreneurship and the connection between the rural industries on the basis of factor resources flow are related. As a result, this study analyzed the practical situation of migrant workers' return-home entrepreneurship. Next, it used a combination forecast method and dynamic simulation model of the migrant workers' return-home entrepreneurship and revitalization of the relationship with the rural industry. The resuscitation of rural industries can be efficiently facilitated by the return of rural migrant workers to launch firms. Their success can attract more migrant workers to launch businesses. The total regeneration of rural areas and the prosperity of farmers can be achieved by effectively linking the return of migrant workers to launch businesses and the revival of rural industries.

## 1. Introduction

The rural rejuvenation strategy was presented in the 19th CPC National Congress report. The Strategic Plan for Rural Regeneration (2018–2022) was released by the CPC Central Committee and the State Council. It outlined a strategy for rural revitalization. Farmers play a crucial role and are the central focus of the rural revival strategy. Given the general demographic makeup of rural areas and the rural labor surplus, many agricultural workers migrate to cities yearly. The remaining rural workforce are generally older and less educated mainly because of the more traditional agricultural production and management. Therefore, agricultural modernization needs to rely on the existing rural labor force. Agricultural workers must be moved to towns and villages. In order to strengthen support for the revitalization of rural talents in China, the No. 1 document of the Central Government pointed out that it should be widely used to train new professional farmers, train professional talents, and make full use of scientific and technological talents. In order to promote “returning home,” China has taken various measures to greatly increase farmers' enthusiasm to return home. Currently, many farmers participate in home-related entrepreneurial activities, and a new type of professional farmers is growing rapidly. Agricultural production is supported by the input of science and technology, personnel, and capital. In the key stage of building a well-off society in an all-round way, it is of great significance to formulate the rural reconstruction strategy to eliminate poverty and solve some employment problems. Accelerating the process of agricultural modernization, developing and expanding rural agricultural industry and culture, and improving the living conditions of rural residents positively influence the implementation of the agricultural revitalization strategy. The entrepreneurial spirit of migrant workers is more complex. In other words, there are an emotional attachment to rural areas and a desire to increase family income, self-esteem, and other spiritual sustenance. Migrant workers believe that returning home to start businesses can not only fulfill the responsibility of taking care of the elderly and children, but also save unnecessary travel costs, which is a necessary choice in line with rational economic laws [1].

Combining many distinct forecasting techniques for the same topic is known as combined forecasting. It combines various quantitative and qualitative procedures and several quantitative ones. More significantly, it combines quantity and quality in practice [2]. The combined forecasting method offers certain benefits when looking at the mechanism linking migrant workers who return home to launch their own firms with the rehabilitation of rural industries. This study dynamically simulated the relationship between migrant workers who return home to start their own businesses and industrial revitalization and rejuvenation based on the interpretation of the current situation of migrant workers returning home to start their own businesses, combined with previous research and theoretical knowledge and combination prediction, using linear prediction combination. The study discovered that the link between migrant workers returning home to start businesses and the revitalization of rural industries is based on the flow of resources and is inextricably linked to human, economic, and social resources. Finally, it is concluded that we should lay a good foundation for rural industry revitalization, promote the system's balanced operation, enrich the main body, strengthen the interconnection, improve the mechanism, optimize the interaction of rural migrant workers, integrate high-quality resources, and constantly promote the implementation of the agriculture revitalization strategy. This study systematically analyzes the problems in the linkage process and puts forward some effective solutions and suggestions, which are conducive to the successful entrepreneurship of the returning migrant workers and attract more migrant workers to return to their hometowns to realize the prosperity of rural industry and the common prosperity of farmers.

## 2. Research Background

### 2.1. Overview of Combinatorial Prediction Algorithms

Bates and Granger were the first to propose the concept of combinatorial forecasting. Their starting point was an understanding of the challenges involved in creating realistic models. In order to address the issue of a single regression prediction model's weak compatibility, the combined prediction model can present information using a single prediction model and establish a more accurate and stable prediction model using the appropriate combination model *via* the weight coefficient and different methods. Portfolio forecasting is one of the most important forecasting research methods, which is widely used in business, society, and management [3].

When designing a combined prediction model, two aspects should be considered: the selection of the prediction model and the distribution of the model's weight [4]. Select individual forecasting models. Other researchers have analyzed the choice of a single forecasting model from qualitative and quantitative perspectives. From the qualitative point of view, according to the principles of applicability, diversity, and cost, the individual forecast model is considered; the appropriate individual forecast model is selected; and the objective, the nature of the forecast change, and the applicability of the individual forecast model are analyzed. The method of determining the number and weight of a single prediction model is also proposed [5]. The combined prediction model based on the difference algorithm is compared to the prediction model composed of the first prediction algorithm with the most effective prediction effect [6] by studying the difference algorithm and using it to select subprojects. The experimental results show that the model offset algorithm improves the combined model's prediction accuracy and approximate weight distribution. It is proposed to use a combined prediction model based on interval accuracy and the IOWA operator. If the value of the lens function is not differentiable, the weight is changed based on the shape. An example demonstrates the efficacy of combined forecasting. Zhang Yite applied the combined prediction algorithm to the fault prediction of the train control device and proposed a combined prediction method based on the cross-entropy theory [7]. Firstly, the *K*-nearest neighbor nonparametric regression model and the improved grey Ehrman neural network model are used for prediction analysis. Then, the cross-entropy theory is used for optimization, and the typical equilibrium model is selected for comparative analysis. Chen Yi studied the short-term combination prediction of electric power and wind farm, established the combination prediction model based on the optimal combination prediction model and the nonoptimal combination prediction model using the microselection algorithm model, and made a comparative analysis. Finally, it is concluded that, under the optimal combination, the combination prediction model selected by the algorithm is better than the nonoptimal combination prediction model [8]. Zhou Li used the entropy theory and ant colony algorithm to construct the weight of the combined prediction algorithm. Yu Jin mixed the flow prediction model based on the theory of the intelligent is studied. Based on the introduction of forecasting models based on gray system theory, the advantages of the combined forecasting model are investigated, and a combination of different algorithms to optimize prediction algorithm. On the basis of the combination model, the combination model of the agricultural product recommendation price algorithm is established, which improves the prediction accuracy and makes the algorithm more stable. According to the prediction theory based on reconstruction, a model combining the combined prediction method with the empirical decomposition model, least squares vector mechanism support, and the moving average model of the autoregressive integration was proposed [9].

### 2.2. Research Review of Migrant Workers Returning Home to Start Businesses and Rural Industry Revitalization

A literature survey on migrant workers returning to start businesses was conducted in the early 1990s [10]. In 1994, the phrase “migrant workers returning home to start businesses” appeared in a national policy document for the first time. Scientific research shows a general trend from fragmentation to systematization compared to agricultural enterprise practices and policies. Related scientific studies can be divided into three categories: studies show that entrepreneurship has become one of the career choices of agricultural workers at the beginning of this century and the number of migrant workers increases at an annual rate of about 7% [11]. Entrepreneurship is an economic activity, which accurately identifies potential according to industry development, market conditions, and existing equipment and excavates potential to integrate resources and large-scale marketing to create value and achieve greater returns. Due to the idiosyncratic nature of personal devices, the entrepreneurial spirit of agricultural workers is complicated by their decision-making based on economic rationality and emotional attachment, such as emotional attachment to rural areas, desire to increase family income, and self-esteem [12]. Some scholars argue that returning farmers rely on resources and skills acquired through professional experience, a problem facing the countryside in recent years. Under the influence of reason and emotion, the farmers decided to return to their villages to do business. Compared with the past, the Communist Party Central Committee has reached a new level in helping farmers start their own businesses. A series of central documents emphasize that promoting the return of farmers to their hometowns should be one of the priorities. In addition, as a major group, the new generation of migrant workers have broader horizons, more solid knowledge and skill base, and a long-term perspective compared to their parents. They have accumulated sufficient capital, valuable work experience, coordination, and cooperation skills, and the interpersonal network with close ties to the industry forms the real foundation for their entrepreneurship in China. Some scholars define the returning rural workers as those leaving the counties and cities or being laid off due to a variety of measures: lack of knowledge and resources such as prerequisites for entrepreneurship, home, or large-scale development of the small and medium-sized city agriculture. This is the inevitable choice of migrant workers under the law of economic rationality [13]. Some scholars have studied the role of returning rural workers in starting businesses from the macro-level and analyzed the source of returning entrepreneurial elites, the new model of rural reconstruction, and the entrepreneurial management mechanism. The growth of entrepreneurial spirit among returning farmers increases household income, thus stimulating consumption. It stimulates high-quality economic development, promotes the structural transformation of supply chains, and reduces the overall cost of social governance. This will also help in the lagging population's employment rate and quality of life [14]. Other academics believe that migrant workers returning home to start businesses can hasten the accumulation of labor force and production factors in small cities, propel the development of small and medium-sized cities and local economic growth, and play a positive role in the social and economic field. The transfer of the local labor force creates new opportunities for large-scale employment and stable rural income growth. Returning home to start a business can help develop the rural economy and improve the local civilization level, adapt to the structure of the agricultural sector, set an example for the surrounding residents, and solve the problem of abandoned children and shanty towns caused by social problems. Macro and micro factors influence the return of migrant workers to start businesses. Some researchers have examined the macro-level differences in development and living conditions between urban and rural areas and the entrepreneurial decisions made and supported by urban and rural areas. Cao believed that, with the improvement of the business environment, the effective implementation of preferential agricultural policies, the gradual attention paid to social issues such as childcare, and the more comprehensive measures to ensure the success of entrepreneurship, the demonstration effect of agricultural workers is the main motivation for agricultural workers to start new businesses at this stage [15]. In addition, this study focuses on the individual characteristics of agricultural workers, risk factors, capital factors, self-actualization, work experience, and loan facilities and analyzes the behavior and readiness of farmers to return home at the micro level. Other scholars conducted a series of discussions on the practical difficulties faced by returning farmers and identified five bottleneck problems: financing, project selection, land use, recruitment, and work difficulties. In addition, rural and handicrafts industries have low entrepreneurial enthusiasm, weak awareness of cooperatives, low level of entrepreneurial departments, low awareness of preferential policies, and weak ability to obtain resources, which result in a low success rate of operation and a real dilemma in industrial development [16].

## 3. Research Methods and Materials

### 3.1. Basic Theory

#### 3.1.1. Combined Forecasting

*(1) Concept*. A forecasting method based on two or more different forecasting methods for the same problem is known as combined forecasting. It is a combination of several quantitative and qualitative methods. More importantly, it combines both quality and quantity in practice. The primary goal of the combined forecasting methods is to maximize prediction accuracy by combining information from various methods. For example, it is difficult to develop a single forecasting model that is very close to the reality of frequent macroeconomic fluctuations and provide a stable and consistent explanation of their causes during the transition to a market economy. Theoretical and practical studies show that the combined forecasting model can be more predictive than any single predictor for different forecasting models and data sources. The prediction model can reduce systematic errors and increase prediction efficiency [17].

**Figure 1:**
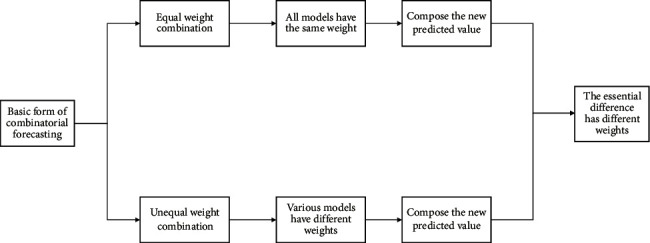
Basic form of combination prediction.

*(2) Basic Form*. There are two basic forms of combined forecasting: 1) balanced combination refers to combining the prediction results of different forecasting methods into new prediction results according to the same weight array and 2) nonuniform combination is the prediction of a combination based on different weight numbers. Other than that, the principles and methods of application of these two forms are consistent, but there are some differences in the definition of weight. The combined prediction of the nonuniform combination is relatively accurate in terms of prediction [18] as shown in Figure 1.

*(3) Application Principles and General Steps*. Application principle: the combination of qualitative and quantitative analyses [19].

**Figure 2:**
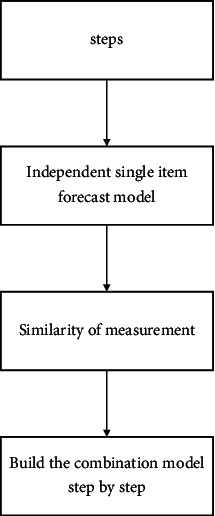
General steps for combination prediction.

Steps: take economic forecasting as an example; the general step is to build a separate individual forecasting model based on economic theory and practice. Model similarity was measured by systematic cluster analysis. According to the cluster analysis results, a combined prediction model is established [20]. The steps are shown in Figure 2.

**Figure 3:**
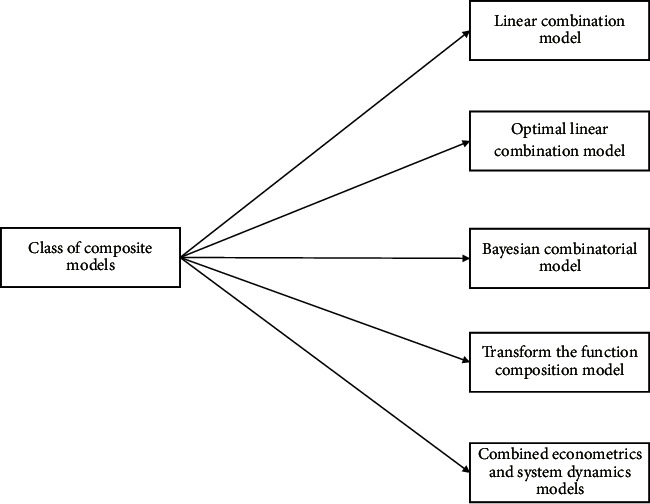
Category of combination prediction.

*(4) Types of Combination Models*. Model 1: linear combination model; model 2: optimal linear combination model; model 3: Bayesian combination model; model 4: transformation function combination model; model 5: combination model of econometrics and system dynamics, as shown in Figure 3.

#### 3.1.2. Migrant Workers Return Home to Start Businesses

The lower cost of living and the desire for a family reunion are motives force of migrant workers to return home. The improvement of urban migrant workers' wages, living conditions, and cultural surroundings is the power to attract agricultural workers into the city, but the unequal social public service system has made migrant workers realize that their living conditions have not improved in a groundbreaking way. As a result, they are encouraged to return home for productive activities that are expected to increase their income. The domestic environment provides policy support for the development of migrant workers returning home, and the rise of the “entrepreneurship boom” has invigorated migrant workers returning home to start businesses.

### 3.2. Main Formulas and Processes

(1) Building a linear combination model: the linkage approach of migrant workers returning to their hometowns to start their own business and rural industrial revitalization is mainly a combination of human resources, economic resources, and social resources, so as to find the best solution:
  (1) $$\begin{array}{c} Z_{1}=8.9+0.61x_{1}+0.21x_{2}+0.18x_{3}\text{,}\\ Z_{2}=5.6+0.53x_{4}+0.47x_{5}\text{,}\\ Z_{3}=6.4+0.74x_{6}+0.36x_{7}\text{.} \end{array}$$
In the above formula, *Z* denotes rural industry revitalization; *x*_1_, *x*_2_, *x*_3_represent the human resources of migrant workers returning home to start businesses, which are the number, education background, and age of migrant workers; *x*_4_, *x*_5_ represent the economic resources of migrant workers returning home to start businesses, which are their own economic strength and bank loan policy; and *x*_6_, *x*_7_ represent the social resources of migrant workers returning home to start their own businesses, which are respectively the entrepreneurship situation and national macro policies.
(2) Optimization of the combination model:
  (2) $$\begin{array}{c} M_{i}=\frac{\overset{n}{\underset{i=1,2,3}{\sum }}Z_{i}-Z_{i}}{\overset{n}{\underset{i=1,2,3}{\sum }}Z_{i}}\frac{1}{n-1}\text{.} \end{array}$$
In the above formula, *M*_*i*_ represents the revitalization of the optimized rural industry.
Based on the above formula, the linkage between migrant workers returning home and rural industry revitalization is based on their human, economic, and social resources.

## 4. Results and Discussion

### 4.1. Main Analysis

Based on the human, physical, and social capital of migrant workers returning home to start businesses, this chapter examines their relationship with rural industry revitalization. The human, material, and social capital of migrant workers who return home to start businesses are examined in this study.

#### 4.1.1. Analysis of Human Capital of Migrant Workers Returning Home

**Figure 4:**
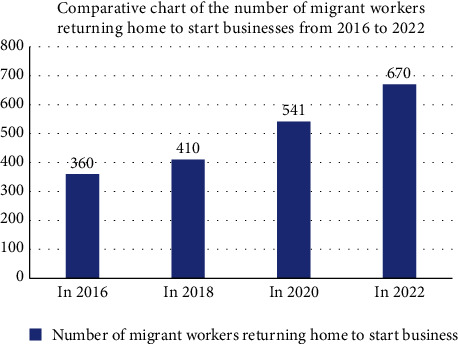
Number of migrant workers returning home to start businesses from 2016 to 2022.

The human resources analysis of migrant workers returning home to start businesses includes information such as the number of migrant workers, educational background, age, and other factors. It is clear from the analysis and discussion that (1) year after year, the number of migrant workers returning home to start businesses grows. The key figures are as follows: in 2016, 3.6 million migrant workers returned home to start businesses, 4.1 million in 2018, 5.41 million in 2020, and 6.7 million in 2022. According to data analysis, the number of migrant workers returning home to start businesses is increasing year after year. The primary reason for this is that national policy supports rural industry policy. More migrant workers are willing to join the ranks of the rural industry revitalization, which provides entrepreneurial vitality to the rural industry revitalization, as shown in Figure 4.

**Figure 5:**
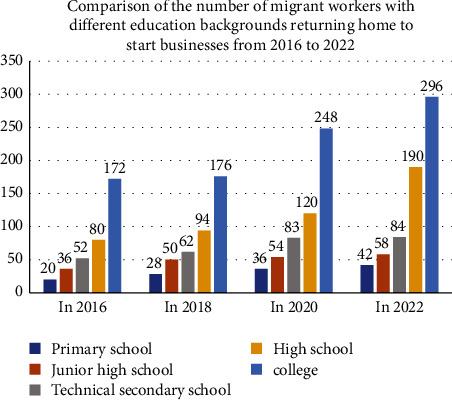
Number of migrant workers with different educational backgrounds returning home to start businesses from 2016 to 2022.

**Figure 6:**
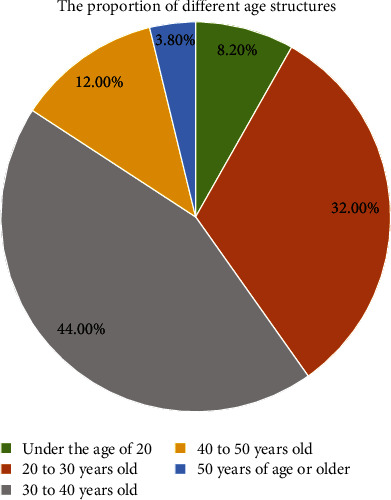
Proportion of migrant workers returning home to start businesses in different age groups.

(2) Those who have received higher education, such as junior colleges, have the most educational background among migrant workers returning home to start businesses. The key figures are as follows: in 2016, migrant workers returning home to start businesses had an educational background of 200,000 in primary schools, 360,000 in junior middle schools, 520,000 in technical secondary schools, 800,000 in senior high schools, and 1.72 million in junior colleges. In 2018, there are 280,000 in primary schools, 500,000 in middle schools, 620,000 in technical secondary schools, 940,000 in high schools, and 1.76 million in junior colleges. In 2020, there are 360,000 in primary schools, 540,000 in middle schools, 830,000 in technical secondary schools, 1.2 million in high schools, and 2.48 million in junior colleges. In 2022, there will be 420,000 primary school graduates, 580,000 junior high school graduates, 840,000 technical secondary school graduates, 1.9 million senior high school graduates, and 2.96 million junior college graduates. According to the data analysis, the following education levels are most common among migrant workers returning home to start businesses: junior college, high school, technical secondary school, junior high school, and primary school. The number of rural migrant workers with various degrees of entrepreneurship is increasing year by year, but with lower degrees, such as elementary and junior high school; compared to high school, college, and other relative degree higher people, the quantity is small. Therefore, better education of migrant workers makes them more willing to start a home business, and the rural industries provide good human resource reserves, as shown in Figure 5.

(3) The age group of migrant workers returning home to start businesses is mainly 30–40 years. The main data are as follows: the age structure under 20 years accounts for 8.2%, the age structure of 20–30 years accounts for 32%, the age structure of 30–40 years accounts for 44%, the age structure of 40–50 years accounts for 12%, and the age structure over 50 years accounts for 3.8%. According to the data analysis, most of the age groups of migrant workers returning home are between 20 and 40 years. It has a great advantage in starting a business. It has certain advantages in terms of age, work experience, and relevant experience, so many people of the age group return to their hometown to start a business, as shown in Figure 6.

#### 4.1.2. Analysis of Material Capital of Migrant Workers Returning Home to Start Businesses

**Figure 7:**
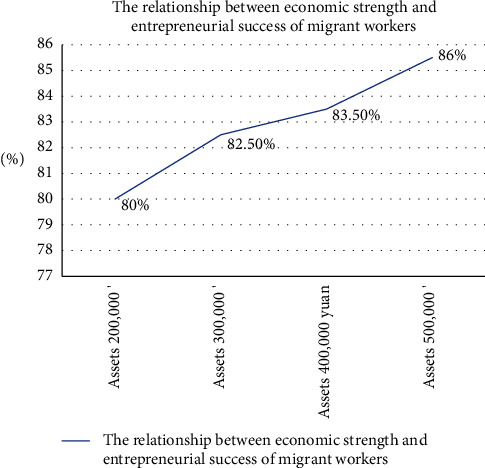
Relationship between economic strength and entrepreneurial success of migrant workers.

**Figure 8:**
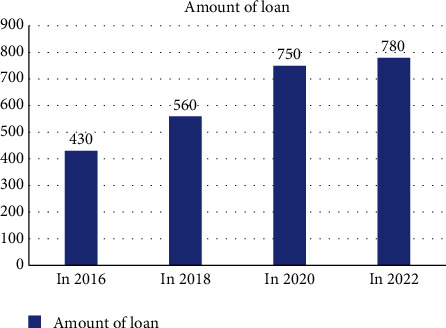
Year-by-year comparison of the amount of bank loans to migrant workers.

The analysis of the economic resources of migrant workers returning home to start businesses includes the economic strength of migrant workers, bank loan policies, and other factors. According to the analysis and discussion, the following can be shown :

1. The economic strength of migrant workers has a great impact on the return of migrant workers to start businesses, which provides a great economic advantage for migrant workers to start businesses. The specific data are as follows: the probability of success of migrant workers with assets of 200,000 yuan is 80%. With assets of 300,000 yuan, the probability of success is 82.5%. With assets of 400,000 yuan, the probability of entrepreneurship success is 83.5%. However, with assets of 500,000 yuan, the probability of entrepreneurship success is 86%. The success of entrepreneurship is closely related to the economic strength of migrant workers themselves. The higher the economic strength, the easier it is to succeed in entrepreneurship, as shown in Figure 7.
2. The bank loan policy also has a great impact on the entrepreneurship of migrant workers, providing material support. In 2016, the bank lent 4.3 million yuan to the entrepreneurship of migrant workers. In 2018, 2020, and 2022, the bank loaned 5.6, 7.5, and 7.8 million yuan, respectively, to migrant workers to help them start their own businesses. According to the data analysis, banks provided more loans to migrant workers to start their own businesses year after year, which encouraged them to start their own businesses and provided them with strong economic support, as shown in Figure 8.

#### 4.1.3. Analysis of Social Capital of Migrant Workers Returning Home to Start Businesses

The analysis of social resources of migrant workers returning home to start businesses includes factors such as entrepreneurship situation and national macro policies. According to the analysis and discussion, the following can be shown:

1. Analysis of the entrepreneurial situation: since the 19th National Congress, the National Party Congress has included the rural revitalization strategy into the work plan and has become the master of the work of promoting agricultural and rural modernization and developing “political leaders” in the new era. Although China still faces insufficient regional development, unbalanced development, and insufficient rural development, from the perspective of development, urban and rural immigration has obvious comprehensive. The city began to absorb various factors of production to achieve rapid development. To realize rural revitalization, we need the support of talents, that is, the unidirectional flow of population from rural areas to urban and rural areas. The startup forms from the big environment; however, rural industries are very good but have the following disadvantages: hindered the development of rural industries, migrant workers exist only blind area in the business, will not be able to properly estimate the risk of entrepreneurship, and more and more people join the startup stage, makes the competition increase, etc., caused the startup form.
2. National macro policy analysis: macro policy proposals and strategic incentives can improve macro policy in the field of migrant workers returning home, initiate a greater incentive effect “from rural to rural,” and fully exploit the role of rural “reserve” to solve the problem of surplus labor. When migrant workers return home to start their own businesses, they should adhere to the fundamental background of rural production, life, and ecology; update and create social networks; highlight development achievements in the process of starting their own businesses; and promote overall rural revitalization. It also reveals the linkage relationship and role of migrant workers returning home to start their own businesses and rural revitalization. It serves as a decision-making reference for relevant government departments to promote the development of the national economy, social and economic development, and the building of a harmonious society.

### 4.2. Results

#### 4.2.1. Linkage Analysis

1. The goal of farmers returning to their hometowns is to revitalize industry, and their return can effectively increase rural resources. More farmers and workers will be able to return home and start their own businesses as rural industries recover. Farmers returning home to start businesses and revitalize rural industries is an important component of rural revitalization. Farmers can start their own businesses again and realize the effective intensification of agriculture. Agricultural industrialization can improve the efficiency of resource allocation, promote the scale and industrialization of traditional agriculture, learn modern agriculture-related technologies, drive the development of modern agriculture, make full use of market opportunities, use internal resources for heterogeneity, and more accurately localize entrepreneurial industries and product types. This will help change the pattern of synchronous flow of high-quality resources from rural areas to cities. This part of the highly skilled human capital with substantial physical capital has become an integral part of the rural reconstruction strategy. According to their comparative advantages in diversification, farmers have accumulated a certain amount of entrepreneurial capital, experience, and technology in the activities of urban management. The rehabilitation and integration of agriculture require substantial support for small and medium-sized enterprises scattered in rural areas, most of which are founded by returning farmers. The existence and development of these enterprises are conducive to the stable employment of surplus agricultural labor force, the steady growth of farmers' family income, the strengthening of collective economic strength, the gradual improvement of rural infrastructure, the continuous improvement of rural public services, and the comprehensive recovery of rural areas.
2. Industrial development and revitalization assist farmers in reestablishing their own businesses, effectively strengthen rural industries, and attract more agricultural workers to establish their own businesses. The revival of rural industries can lay a solid foundation for farmers to return to entrepreneurship. Strengthening basic capacity and sustainable agricultural development can provide incentives and basic support for farmers to return home. Local governments should combine the local social and economic development and the actual allocation of resources and conduct in-depth research on the supply and demand structure of the agricultural products market for those suitable for developing agricultural industries, home to do business. We will strengthen capacity building for sustainable agricultural development and create better business opportunities for returning farmers.

#### 4.2.2. Linkage Analysis

Based on the above analysis of various resources of migrant workers returning home, it is concluded that the development trend of migrant workers returning home to start businesses and rural revitalization is good. However, the linkage mechanism of migrant workers returning home to start businesses and rural revitalization still has the following problems:

1. Insufficient power of linkage mechanism: different types of entrepreneurial industries have different production needs, and the two-way transmission of information up and down and the mechanism of transmission are not perfect, affecting the entrepreneurial quality of entrepreneurs. The discrepancy between rural industrial development and policy supply, and the asymmetry in the demand of migrant workers returning to their hometowns to start their own businesses, leads to more significant fluctuations in the core agricultural industry.
2. There are some problems in the construction of linkage mechanisms, such as imperfect organization and leadership and lack of close interaction between groups. First, the lack of unified organizational management, lack of professional and authoritative organizational leadership, and migrant family entrepreneurs having an unclear understanding of their role in rural revitalization, with their sense of efficacy, accomplishment, pride, and belonging being affected, lead to role and communication barriers in the connection between entrepreneurship and rural revitalization. Second, the lack of interaction between organizations and groups; the lack of platforms for communication, dialog, and cooperation; the lack of the formation of entrepreneurial alliances mainly composed of returning migrant workers; and the imperfect interest linkage mechanism are other factors that hinder the entrepreneurial development of migrant workers.
3. The ability to ensure elements of the linkage mechanism is limited. The most obvious and direct incentive for migrant workers to return home and start a business is capital reward and subsidy. However, the welfare system is limited and only serves a few people. Regional differences in capital reward and subsidy for migrant workers to start a business are due to different financial and development priorities. In addition, the procedure of declaration is complicated and the cycle is long. There is no publicity mechanism around the effect of migrant workers returning home to start businesses. The development of rural industry has strong cyclical characteristics, and migrant workers' land rights and interests are affected to some extent. In comparison to actual needs, the amount, content, and experts of human capital training are insufficient.
4. There are barriers to communication channels for migrant workers returning to their hometowns to start their own businesses, such as inadequate platforms and poor connections; for example, rural resources information cannot be spread or transmitted in a narrow scope, communication ability is weak, market information cannot spread among them, there is two-way information dissemination. In terms of the actual demand for migrant workers returning home to start their own businesses, entrepreneurs' ability to understand relevant policy information is limited in the absence of established channels. Entrepreneurs have limited understanding and interpretation of policy documents due to the lack of comprehensive cultural quality and the ability to use the Internet. The meaning and content of policy documents are not timely spread in the business community, and information dissemination has the nature of delay.

## 5. Conclusion

As the current main body of migrant workers, a new generation of migrant workers has a broader field of vision, a more solid foundation of knowledge and skills of reserves, and a more long-term view. They have accumulated enough capital and valuable work experience and developed the ability of coordination and cooperation. Their industry is closely related to the network. Entrepreneurship provides a realistic basis for them upon return to China. Based on the interpretation of the current situation of migrant workers returning to their hometowns for entrepreneurship, a combination of previous research and theoretical knowledge is used to make predictions, thus dynamically simulating the relationship between migrant workers returning to their hometowns for entrepreneurship and industrial revitalization. The study discovered that the link between migrant workers returning to start businesses and the revitalization of rural industries is based on the flow of resources and is inextricably linked to human, economic, and social resources. Finally, the following findings are reached:

1. Increasing the driving force foundation for industrial development, promoting equal access to facilities, actively building a driving force mechanism for rural industrial development, and striving to combine rural migrant workers returning to their hometowns to start businesses with rural revitalization strategies: we will increase support for migrant workers returning to their hometowns to start businesses; form an infrastructure construction system of “government, entrepreneurs, agricultural, and industrial support”; achieve breakthroughs; resist core areas and key links; and focus on optimizing the business environment based on the dimension of “industry-ecology-culture-ecology-income growth.” We will improve the environment for rural migrant workers in poor counties to return to their hometowns and start businesses. We will fully recognize rural migrant workers in poor counties' positive role in consolidating poverty alleviation achievements and effectively connect them with rural revitalization.
2. Rural characteristic industries should be developed to set the basis for rural entrepreneurship. First, according to market demand and government policies, in combination with the characteristics of local resources and regional conditions, we should cultivate and develop advantageous industries, focusing on developing entrepreneurial spirit based on business. Second, we should perform well in guiding entrepreneurship, play the role of project engine, optimize the layout of the entrepreneurship industry, and lay the foundation for the entrepreneurship market. Finally, we should do a good job in infrastructure security and promote infrastructure equalization. On the basis of improving the infrastructure service system, we will actively attract logistics, ecological and environmental protection, and intelligent Internet big data facilities and establish and improve a modern infrastructure service system. Simultaneously, we will promote the outsourcing of infrastructure construction and public service projects to qualified migrant workers who return home to start their own businesses. Relevant government departments should regularly provide good management, supervision, and follow-up on project implementation.
3. Local relationships underpin the development, transformation, and reconstruction of rural resources. The richness and integrity of the relationship network influence migrant workers' return to start businesses. The participation of the government, businesses, scientific research institutions, and industry associations in rural development can promote the accumulation of human, material, and social resources. As a result, we can enrich the main body, strengthen the entrepreneurial relationship network, and improve farmers' ability to return home to start businesses. The formation of agricultural and industrial enterprise clusters, as well as enterprise clusters with local characteristics, can adapt group behavior to individual behavior and promote individual decision-making and collaborative development. It follows the development model of “migrant workers returning to their hometowns to create industrial clusters + enterprises,” serves as a feedback collector in the group problem interaction process, and forms the mechanism of imitation, demonstration, and adjustment. We should encourage the formation of strategic alliances, encourage migrant workers' return to start businesses, extend to the front and back ends of the industrial chain, and actively build an all-dimensional industrial chain in rural areas. It follows the development model of “migrant workers returning home to start businesses + industry aggregation + government + banks + scientific research institutions + industry associations” and provides “material resources + financial resources + intelligence” services *via* multisubject participation to improve resource cohesion and integration and effectively promote the core competitive advantage industry aggregation of migrant workers returning home to start businesses. The model of “migrant workers returning home to start businesses” provides timely and real-time professional knowledge services *via* the cloud platform and strengthens communication between enterprise groups and government departments, thereby broadening information channels and improving policy information efficiency. We will improve mechanisms, optimize services for rural migrant workers returning to their hometowns to start businesses; encourage the establishment of a “city-county- (district-) town (township),” a three-step management system for farmers returning to their hometowns; establish labor unions for farmers returning to their hometowns; and establish a dialog platform with authorities dealing with the issue of farmers returning to their hometowns to improve the efficacy of those farmers. We will improve the service system and mechanisms for encouraging and training rural migrant workers who return home to start businesses.
4. We will introduce new factors and resources to make entrepreneurship, entrepreneurship bases, and entrepreneurship training bases more inclusive. First, it is necessary to establish an efficient service system for returning rural households and enterprises and improve statistics on entrepreneurship and innovation, multidisciplinary knowledge base, and entrepreneurship consulting platform. We will actively establish the mechanism of party building in colleges and universities and provide various documents, information, and services to farmers returning to their hometowns to start their businesses. Second, we must strengthen financial support, reduce taxes, ensure regulatory accountability, expand credit support, and increase financing channels for returning entrepreneurs. It is necessary to establish a risk prevention mechanism for returning farmers, comprehensively solve the problem of failure in returning to their hometown, and actively investigate the problem of setting up a commercial insurance company for returning farmers. Thirdly, based on the training needs of entrepreneurs, we should improve training coverage, broaden the training content, expand the training form, and improve the quality of the training team. We should actively adopt the “tutorial system” mode to enhance the pertinence of entrepreneurship training for migrant workers. Fourth, we will strengthen the guarantee of water and electricity for rural migrant workers returning home.
5. In the process of establishing the linkage mechanism between migrant workers returning home to start businesses and rural revitalization, we should give full play to the enthusiasm of returning migrant workers and focus on providing them with statistical data services and communication platform construction services: 1) the statistics of market demand data and the establishment of an entrepreneurial guidance mechanism, 2) policy-oriented statistical information to enhance the universality of policies, 3) statistics of entrepreneurial service information and optimization of the entrepreneurial environment, 4) establishment of multisectoral and multifunctional collaboration mechanisms to facilitate more effective pooling of resources by broadening the range of actors, and 5) a talent pool to enhance the minds of farmers to start businesses back home.
6. We should build high-quality resources for rural, human resource security, market supervision, and other departments; create a cloud platform for migrant workers to return home and start businesses; improve information exchange between departments; promote the effective integration of all departments' resources; and provide timely feedback to relevant subjects. We will work together to establish an agricultural and industrial enterprise base, mobilize resources for projects in the agricultural and industrial complex's location, and promote business environment structure optimization. The association, comprising scientific research institutions, universities, businesses, and entrepreneurial migrant workers, will be in charge of disseminating information, teaching entrepreneurial skills, and sharing entrepreneurial experiences. We will encourage private capital and government departments to collaborate in establishing angel investment funds for migrant workers returning home to start businesses, expanding channels for entrepreneurs to obtain venture capital, promoting top-level design for migrant workers returning home to start businesses, reducing the blindness of starting businesses, and promoting rural revitalization. Rural migrant workers who return to their hometowns to start businesses are encouraged to collaborate with consumers to establish production bases, and a “community industry” model is adopted, in which consumers take communities as units and subsidize entrepreneurs, and production bases are managed by entrepreneurs to provide final products. We will create a mechanism to connect the interests of migrant workers and local farmers and promote the joint development and sharing of rural industries.

## Data Availability

The dataset is available upon request.
